# Wind-current feedback is an energy sink for oceanic internal waves

**DOI:** 10.1038/s41598-023-32909-6

**Published:** 2023-04-11

**Authors:** Audrey Delpech, Roy Barkan, Lionel Renault, James McWilliams, Oladeji Q. Siyanbola, Maarten C. Buijsman, Brian K. Arbic

**Affiliations:** 1 Department of Atmospheric and Oceanic Sciences, UCLA, Los Angeles, USA; 2 Department of Geophysics, Tel-Aviv University, Tel-Aviv, Israel; 3 LEGOS (Laboratoire d’Etudes en Géophysique et Océanographie Spatiales), Toulouse, France; 4 School of Ocean Science and Engineering, Stennis Space Center, University of Southern Mississipi, Hattiesburg, USA; 5 Department of Earth and Environmental Sciences, University of Michigan, Ann Arbor, USA

**Keywords:** Physical oceanography, Atmospheric dynamics

## Abstract

Internal waves contain a large amount of energy in the ocean and are an important source of turbulent mixing. Ocean mixing is relevant for climate because it drives vertical transport of water, heat, carbon and other tracers. Understanding the life cycle of internal waves, from generation to dissipation, is therefore important for improving the representation of ocean mixing in climate models. Here, we provide evidence from a regional realistic numerical simulation in the northeastern Pacific that the wind can play an important role in damping internal waves through current feedback. This results in a reduction of 67% of wind power input at near-inertial frequencies in the region of study. Wind-current feedback also provides a net energy sink for internal tides, removing energy at a rate of 0.2 mW/m$^2$ on average, corresponding to 8% of the local internal tide generation at the Mendocino ridge. The temporal variability and modal distribution of this energy sink are also investigated.

## Introduction

The ocean circulation is forced at large scales by boundary fluxes of momentum, heat and freshwater as well as by the astronomical tidal potential. The variability of these forcings provide energy to the ocean, whose motions span a wide range of spatial and temporal scales. Yet, climate equilibrium can only be reached through the dissipation of these energy sources. In order to understand the ocean response to future climate scenarios, the routes to dissipation must be understood and quantified.

One route to mixing in the ocean interior is the breaking of internal waves^1^. Internal waves (IWs) represent indeed a large energy reservoir. At a global scale, about 1TW is converted from astronomically forced barotropic tides into tidal internal waves, also called internal tides (ITs)^2–4^. This conversion occurs mainly at oceanic ridges and seamounts. In addition, 0.3–1.4 TW is converted into near-inertial internal waves (NIWs) from high-frequency wind forcing^5–7^. From their generation, IWs exchange and redistribute energy across scales through different processes such as wave–wave interactions^8,9^ and eddy–wave interactions^10–16^. When the energy reaches small enough scales, mixing occurs through instabilities.

IWs cannot be resolved in the current state-of-the-art CMIP-like (Coupled Model Intercomparison Project, Meehl et al.^17^) models used for climate projections, and therefore their effects on the ocean global circulation and mean state need to be parameterized. Although the parameterization of IW-driven mixing has been the subject of much research in recent decades, the various estimates rely on an energy balance between generation, divergence of the flux and dissipation^18^, where it is generally assumed that the energy is dissipated in the ocean and is therefore available for ocean mixing. So far, little attention has been paid to the possible role of the winds in damping IW energy.

However, recent studies have shown that the wind can damp energetic oceanic features through current feedback on winds (CFB). At low frequencies, the wind stress provides an energy sink for mesoscale eddies^19–23^ and submesoscale eddies^24^. Recent studies estimate this mesoscale energy sink at a global scale between $\sim$ 28 GW^21^ and $\sim$ 50 GW^23,25^, which represents 20–35% of the total depth-integrated eddy kinetic energy^19,22^. At high frequency, there are only a few studies that focus mainly on near-inertial motions^6,26–29^. The reduction in net energy input in the near-inertial frequency band has been estimated to 0.21TW (corresponding to an average of 60%) at a global scale^28^. To our knowledge only two studies have reported the effect of CFB at super-inertial frequencies. Flexas et al.^29^ have estimated the kinetic energy fluxes from semi-diurnal and higher frequencies to vary between 0 and − 0.9 mW/m$^2$ in three different regions of the globe (Kurushio, Kergelen region and north-eastearn Atlantic). But this needs to be corroborated by other models and the underlying processes deserve further investigations. In particular, the formulation of the stress used in all of these studies does not take into account the partial reenergization of the wind that would occur in a coupled model^22^. Renault and Marchesiello^30^ have shown in a coupled simulation of the English channel that the tidal currents can drag the atmosphere and generate a tidal low-level wind, implying a negative kinetic energy flux at the semi-diurnal frequency. This coupling has been attributed to the barotropic tides, which have a strong velocity signature on continental shelfs such as the English channel. The effect of the winds on the ITs is however not discussed.

The increasing spatial and temporal resolution of oceanic models makes it possible to investigate these interactions at higher frequencies and in particular for IWs. In this study, we propose to further investigate the effect of the CFB on IWs with a focus on the internal tides. We will answer the following questions: Can the IWs exchange energy with the atmosphere? How significant is this exchange and how does it affect the energetics of IWs? Which mechanisms control the variability of these energy exchanges? What are the underlying physical processes?

**Figure 1:**
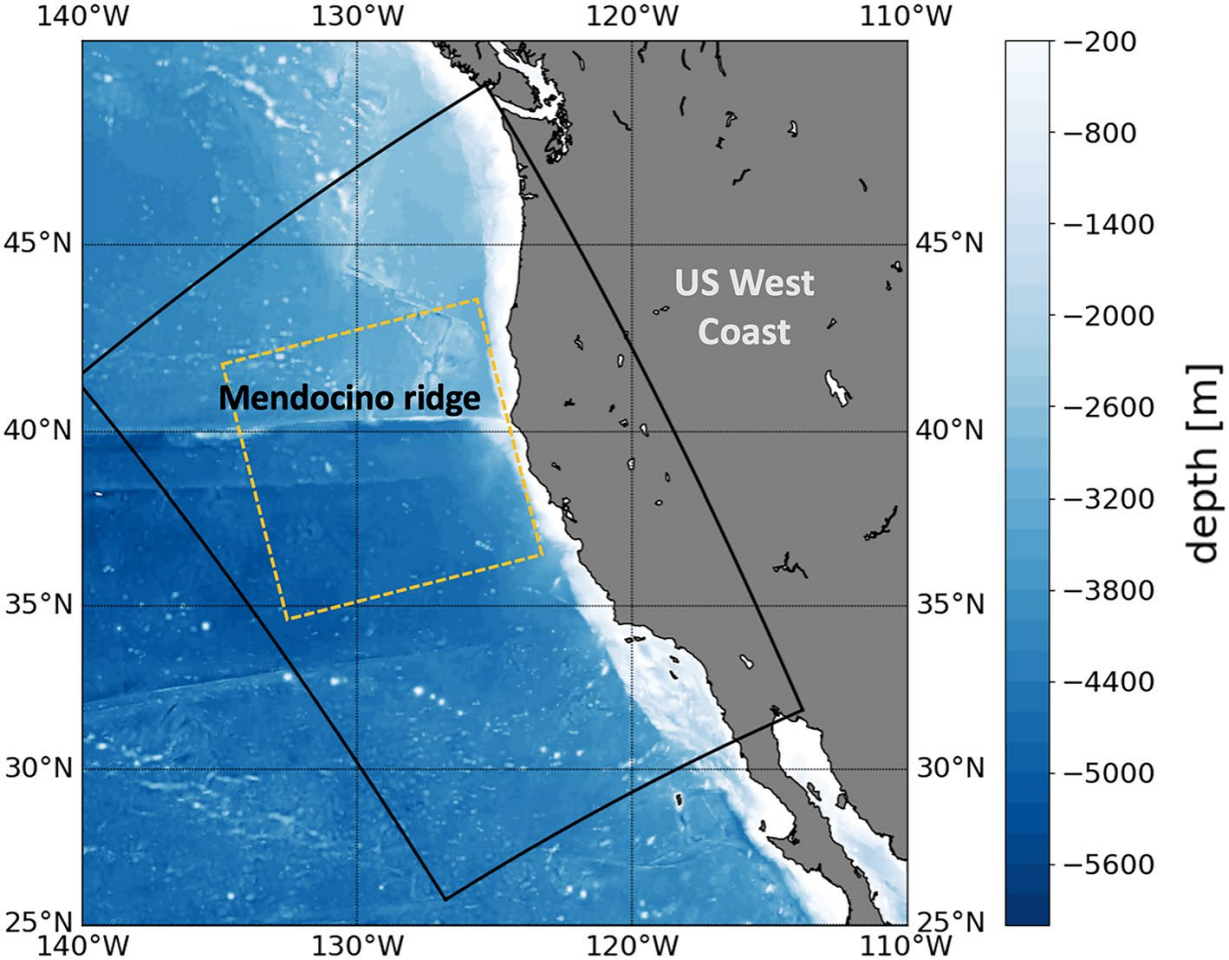
Simulation domain (black) along the US West Coast in the Northeastern Pacific. The yellow dashed box indicates the region used to compute the co-spectrum.

We investigate these questions using a regional numerical simulation of the northeastern Pacific ocean along the US West coast (USWC). The simulation region is shown on Fig. 1. The simulation is performed using the CROCO (Coastal and Regional Ocean COmmunity) model^31–33^. It is forced at the open boundaries with sea surface height, temperature, salinity and velocity from a coarser simulation at an hourly resolution. The internal tides at the boundaries have been generated by the HYbrid Coordinate Ocean Model (HYCOM, Bleck et al.^34^) that include simultaneous tidal and atmospheric forcing^35–37^. These simulations have been extensively validated by Renault et al.^38^ for the low-frequency part and by Siyanbola et al.^39^ for the high-frequency part. The simulation is forced at the surface by the atmospheric model WRF (Weather Research and Forecasting model, Skamarock and Klemp^40^) with a prescribed wind, air temperature, relative humidity, downward short- and longwave radiations and precipitation rate at an hourly resolution. Fluxes are computed from the prescribed forcings using bulk formulas^41^, with a parameterization of the CFB using the wind stress correction approach of Renault et al.^42^. The simulation has a 2km horizontal grid spacing, is run for a year (October 1st, 2011 to September 30th, 2012) and is forced hourly to allow for the generation and the propagation of locally generated and remotely generated IWs. The region encompasses the Mendocino ridge (see Fig. 1), a local generator of ITs^43^. The barotropic to baroclinic conversion has been estimated to 3GW (7 kWm$^{-1}$), from an extrapolation of local measurements, but may be overestimated^43^. Further details about the simulation are provided in the Method section.

## Results

### Spectral wind power

**Figure 2:**
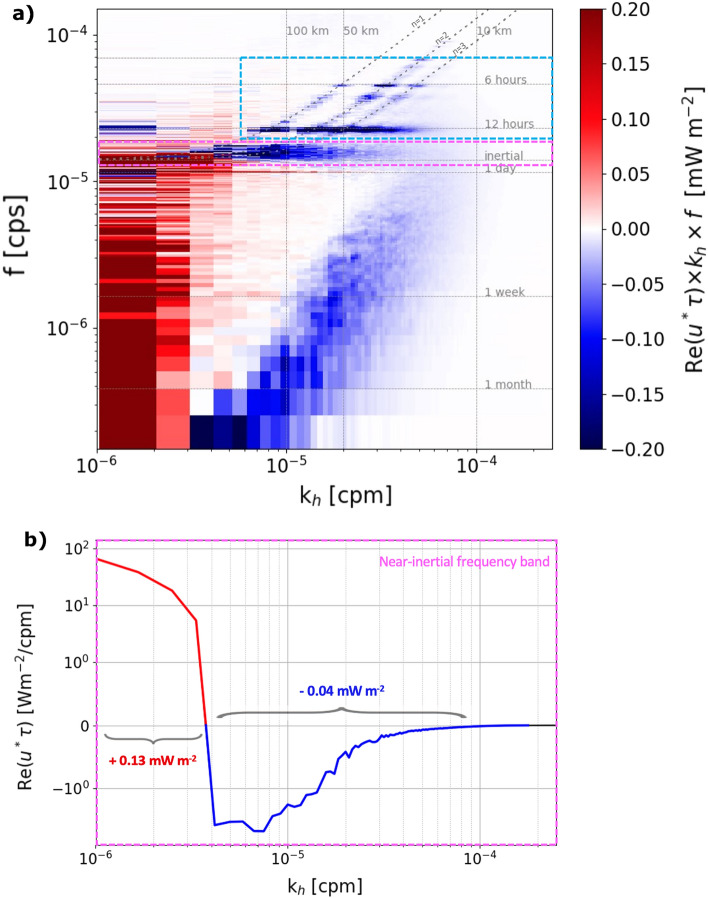
(**a**) Cospectrum of the wind and the surface ocean currents in horizontal wavenumber (x-axis) and frequency (y-axis), computed in the dashed box shown on Fig. 1. Positive values indicate a transfer of energy from the wind into the ocean. Negative values indicate a transfer of energy from the ocean to the wind. The dispersion relations for the first three baroclinic modes (*n* = 1, 2, 3) are indicated. The purple box indicates the limits of the near-inertial frequency band. The blue box indicates the limit of the higher frequency internal wave band. (**b**) Cospectrum of the wind and the surface ocean currents as a function of the horizontal wavenumber integrated in the near-inertial (NI) frequency band 1.39 $\times$ 10$^{-5}$–1.74 $\times$ 10$^{-5}$ cps (periods between 16 and 20 h). The transition scale between NI wind power input and NI wave damping occurs around 5 $\times$ 10$^{-6}$ cpm (200 km).

The cospectrum of wind stress and surface currents quantifies the surface kinetic energy fluxes (Fig. 2a). Negative values indicate the ocean is losing energy to the atmosphere while positive values indicate the converse. The cospectrum of wind stress and surface currents is computed in a square box away from the boundaries (Fig. 1) for the year-long simulation.

Figure 2a shows that ocean-atmosphere energy exchanges occur mainly at the scales for which winds or surface currents are the most energetic (Fig. S2, SI shows the wavenumber-frequency spectra of winds and surface currents). Overall, the wind inputs energy (0.4 mW m$^{-2}$) in the ocean at large scales (> 200 km), in agreement with previous studies^44^. At smaller scales (10–200 km) and low frequencies ( < 1 day$^{-1}$), namely the meso- and submesocale ranges, the wind act as an energy sink for the ocean, removing energy at a rate of − 0.05 mW m$^{-2}$. This phenomenon is known as “eddy killing”^20–22,25^. At smaller scales (10–200 km) and higher frequencies (>1 day$^{-1}$), the wind also act as an energy sink (− 0.2 mW m$^{-2}$). This is particularly so in the near-inertial frequency band, in accordance with previous studies^26,28^. It is also the case at the main tidal harmonics (in particular at the $\sim$ 12 h period corresponding to the M$_2$ semi-diurnal tide and at the $\sim$ 6 h harmonic), and along the dispersion curves for linear waves. Note also the presence of negative wind power at the two main tidal frequencies: diurnal and semi-diurnal at large scale (> 500 km), which correspond to the signature of barotropic tides, consistently with Renault and Marchesiello^30^. This super-inertial “IW killing” (by analogy with the eddy killing) is shown here with an unprecedented remarkable signature.

### The current feedback mechanism

The “IW killing”, which corresponds to a mean negative wind power over IWs (near-inertial, tidal, and high-frequency waves) can be interpreted similarly to the mean negative wind power over an eddy^22^. The underlying conceptual mechanism in both cases relies on the formulation of the wind stress, which is here computed using a bulk formula^41^ and including a wind stress correction to account for the wind response (mimcking coupled models in forced numerical simulations), following Renault et al.^42^:

1 $$\begin{aligned} \varvec{\tau }= \underbrace{\rho _a C_d|{\textbf{u}}_a|{\textbf{u}}_a}_{\text {bulk formula}} \,+ \underbrace{s_{\tau }{\textbf{u}}_c}_{\text {stress corr.}}, \end{aligned}$$

where $\rho _a$ is the surface air density, $C_d$ is the drag coefficient computed following Fairall et al.^41^, ${\textbf{u}}_a$ and ${\textbf{u}}_c$ are the absolute 10 m wind and the surface current vectors and $s_{\tau }$ is the coupling coefficient between surface current and surface stress, $s_{\tau } = - 0.0029|{\textbf{u}}_a|+0.008$ if $|{\textbf{u}}_a| \ge 3$ ms$^{-1}$ and $s_{\tau } = - 0.0007$ Nsm$^{-3}$ if $|{\textbf{u}}_a| < 3$ ms$^{-1}$. Note that $s_{\tau }$ is always negative.

**Figure 3:**
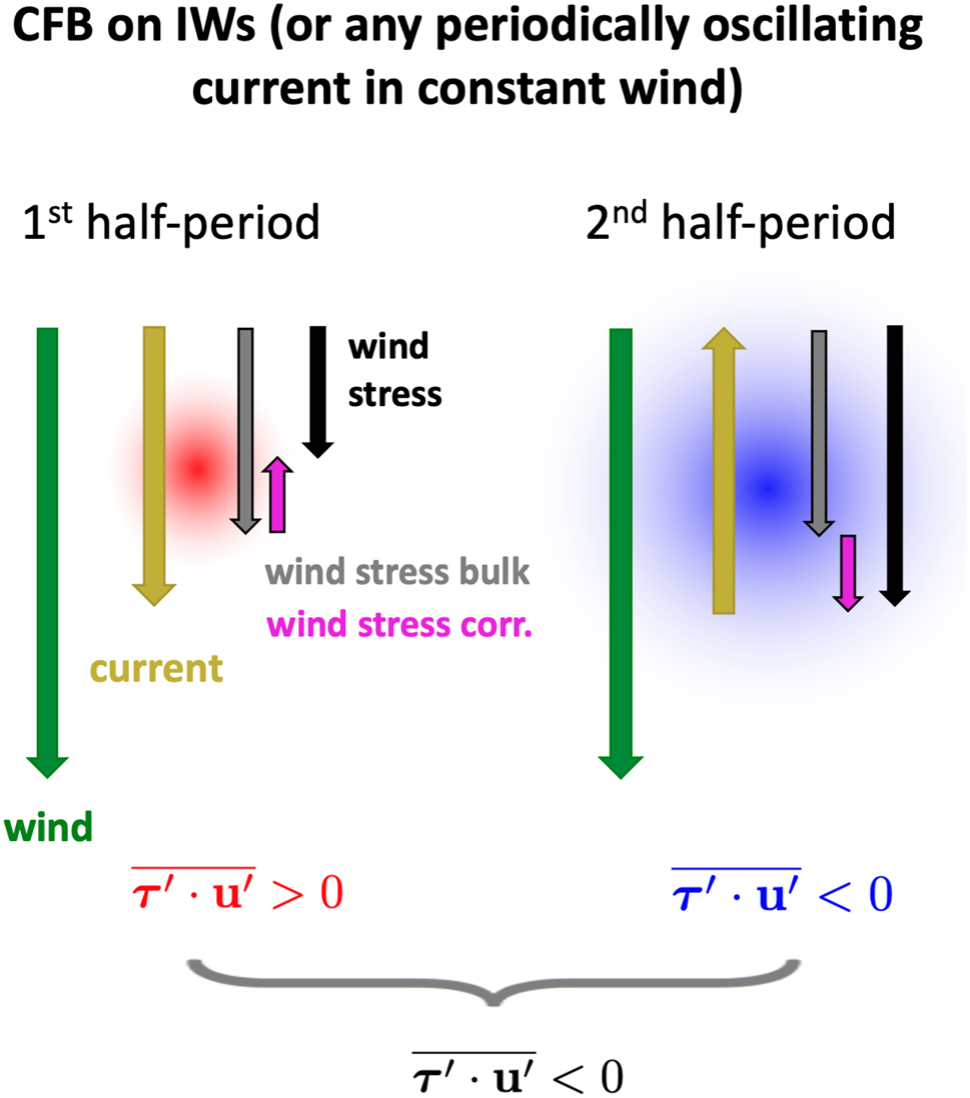
Schematic illustrating the current feedback (CFB) on an internal wave (IW)—or any periodic signal having a surface signature in velocity. The yellow arrows represent the current (the current reverses direction during the second half period of the periodic signal). The green arrows represent the wind, assumed to be constant over an IW period and uniform over an IW wavelength. The black arrows represent the wind stress (Eq. (1)), decomposed between the bulk wind stress (grey) and the wind stress correction (purple). The wind power, corresponding to the inner product of the wind stress and the ocean current, is small and positive (red) when the wind is aligned with the current and large and negative (blue) when the wind is in the opposite direction of the current. This results in a negative wind power, when averaged over a wave period.

In the case of an IW (or any periodic signal with a surface velocity signature) in a constant and uniform wind (or a wind with scales of variation larger than those of the IW), the wind stress (Eq. (1)) is larger during the half-period when the current is flowing against the wind, and smaller during the half-period when the current is flowing in the same direction as the wind. This creates an asymmetry in the wind power. Averaging temporally over a wave period results in a negative wind power (Fig. 3). The assumption of a wind constant and uniform at the scale of IW is realistic given that most of the wind energy is contained at large scales and low frequencies (Fig. S2a, SI).

The main difference between the eddy case and the IW case is that eddies are axisymmetric while linear IWs are generally polarized. This creates a sensitivity of the wind power $\varvec{\tau }\cdot {\textbf{u}}_c$ to the relative direction between the wind and the currents associated with the IWs, the wind power being maximal when the wind is aligned with the currents (Fig. S3, SI).

### Transition scale in near-inertial wind power

Fluctuating winds generate near-inertial oscillations and waves, mainly at the atmospheric synoptic scale, while a damping effect dominates at smaller oceanic scales (Fig. 2). Although several studies have quantified the impact of CFB on the net wind energy input in the near-inertial frequency band, the scale at which the transition occurs has never been determined. Here, we find that the transition scale between positive and negative wind power occurs around 200 km (Fig. 2b). At scales larger than 200 km, the averaged wind power input is 0.13 mW/m$^2$, consistent with Liu et al.^28^ and Alford^6^, who found wind power in the near-inertial band to be $<1$ mW/m$^2$ for this region. At scales smaller than 200 km, the wind removes energy in the near-inertial band at a rate of 0.04 mW/m$^2$.

The total wind power input in the near-inertial frequency band is 0.09 mW/m$^2$. This is a reduction of 67% compared to a simulation that does not parametrize CFB (Fig. S1, SI). This percentage of reduction is consistent with Liu et al.^28^ who estimated a reduction of 60% on a global scale.

### Quantification and variability of the energy sink for super-inertial internal waves

**Figure 4:**
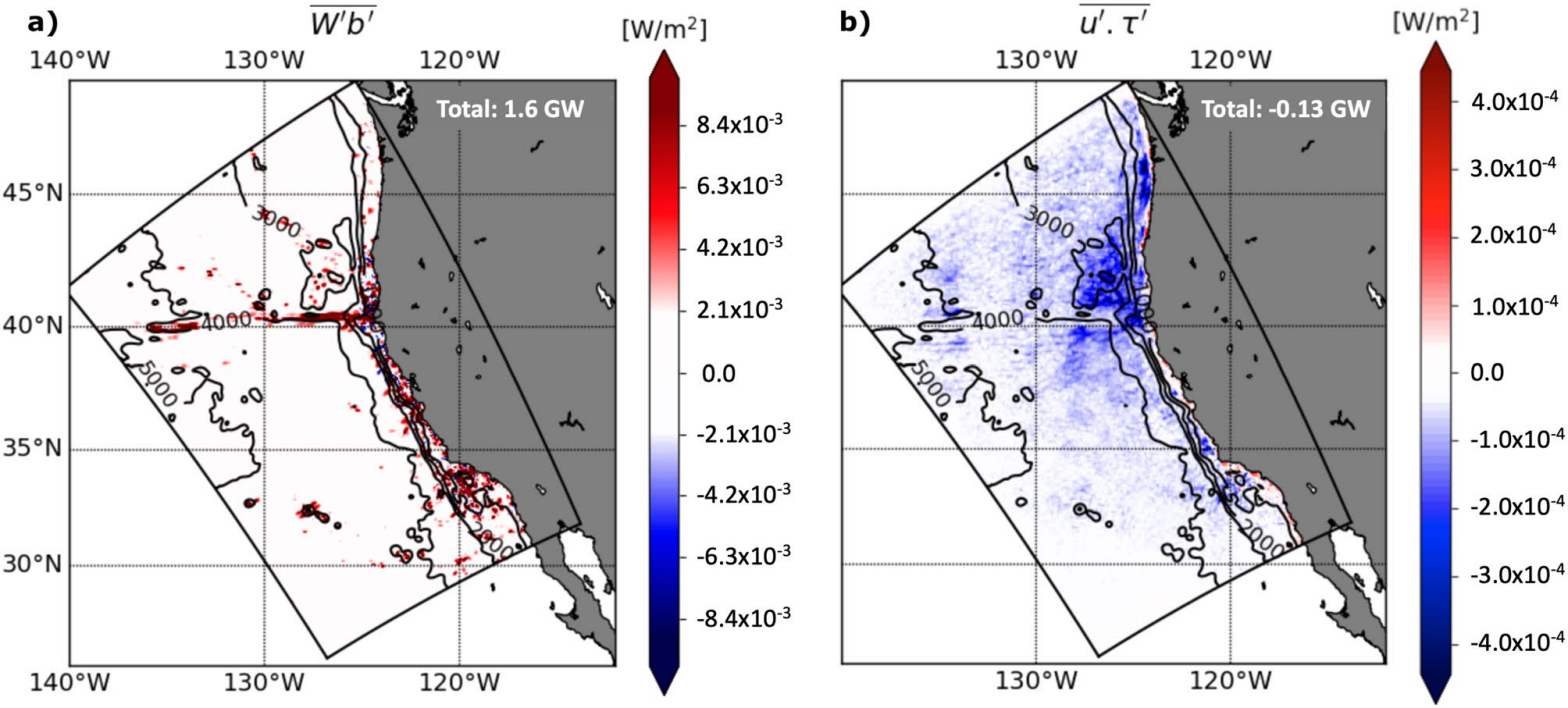
(**a**) Barotropic to baroclinic conversion $\overline{W^\prime b^\prime }$, where the primes correspond to the F-4 h-14 h-180 km filter, corresponding to the blue box in Fig. 2 in spectral space. (**b**) Wind power $\overline{u^\prime \cdot \tau ^\prime }$ on internal tides, where the prime represents the same filtering as in (**a**). Over the region of study, the barotropic tides are converted into internal tide energy at a rate of 1.6 GW and the wind acts as an energy sink for internal tides at a rate of 0.13 GW.

To further investigate the energy sink associated with the “IW killing” at super-inertial frequencies ($> 1.74\times 10^{-5}$ cps), which are mostly dominated by tidal frequencies, we compute the corresponding wind power $\overline{{\textbf{u}}'\cdot \varvec{\tau }'}$, where ${\textbf{u}}$ is the surface horizontal velocity vector and $\varvec{\tau }$ is the wind stress vector. We compare this power with the energy source for internal tides given by the barotropic to baroclinic conversion $\overline{\int _{-H}^\eta W'b'dz}$, where *W* is the barotropic vertical velocity, *b* is the buoyancy anomaly, $-H$ is the depth of the ocean and $\eta$ the position of the free surface relative to the mean sea level (Fig. 4). Here, the overbars correspond to a one-year average and the primes correspond in both cases to a combination of a temporal Butterworth band-pass filter with cutoff periods at 4 and 14 h, and a spatially uniform highpass filter with a cutoff scale of 180 km, referred to as F-4 h-14 h-180 km in what follows. This filter has a spectral footprint shown by the blue box in Fig. 2. Note that the cutoff length scale is above the first baroclinic mode wavelength ($\sim$ 160 km), that the filter used is relatively sharp in spectral space and that the absolute values of conversion and wind power have been found to be slightly sensitive to the choice of the filter scales, but that the ratio between them is not (not shown).

Over the entire simulation region, 1.6 GW is converted from barotropic to baroclinic tides (Fig. 4a). The conversion occurs mostly at the Mendocino ridge (at the westernmost and easternmost sides, which are supercritical to M$_2$ IWs) and at specific locations along the shelf as in the Southern California Bight, as expected from previous studies^43,45^. From this 1.6 GW, 0.13 GW is dissipated by the negative wind power on average (Fig. 4b), representing a reduction of 8%. Most of this energy loss due to wind power occurs along the energy beam radiating from the Mendocino ridge, where it reaches 0.4 mW m$^{-2}$ (Fig. 4b). This energy loss is one order of magnitude less than the mesoscale kinetic energy flux in western boundary currents^46,47^ and in the Antarctic Circumpolar Current (− 4 mW m$^{-2}$^25^), but is comparable to the mesoscale kinetic energy flux over the California Current, which were estimated to be $\sim$ 1 mW m$^{-2}$^22,25^. Flexas et al.^29^ reported semidiurnal and higher frequency energy flux ranging from − 0.1 to -10 mW m$^{-2}$ at three different sites and our estimate at a fourth site lies within that range. The spatial distribution of the negative wind power over the USWC region (Fig. 4b) looks very similar to the spatial distribution of mode 2 and 3 internal tides beams signature in surface KE and is less similar to mode 1 surface KE distribution (Fig. S6, SI). This spatial pattern will be further discussed in the next Sections.

The estimated wind power of − 0.13 GW over the California current region is an averaged estimate. The wind power has in reality some spatial and temporal variability, which are further investigated in the next two sections.

#### Seasonal variability

**Figure 5:**
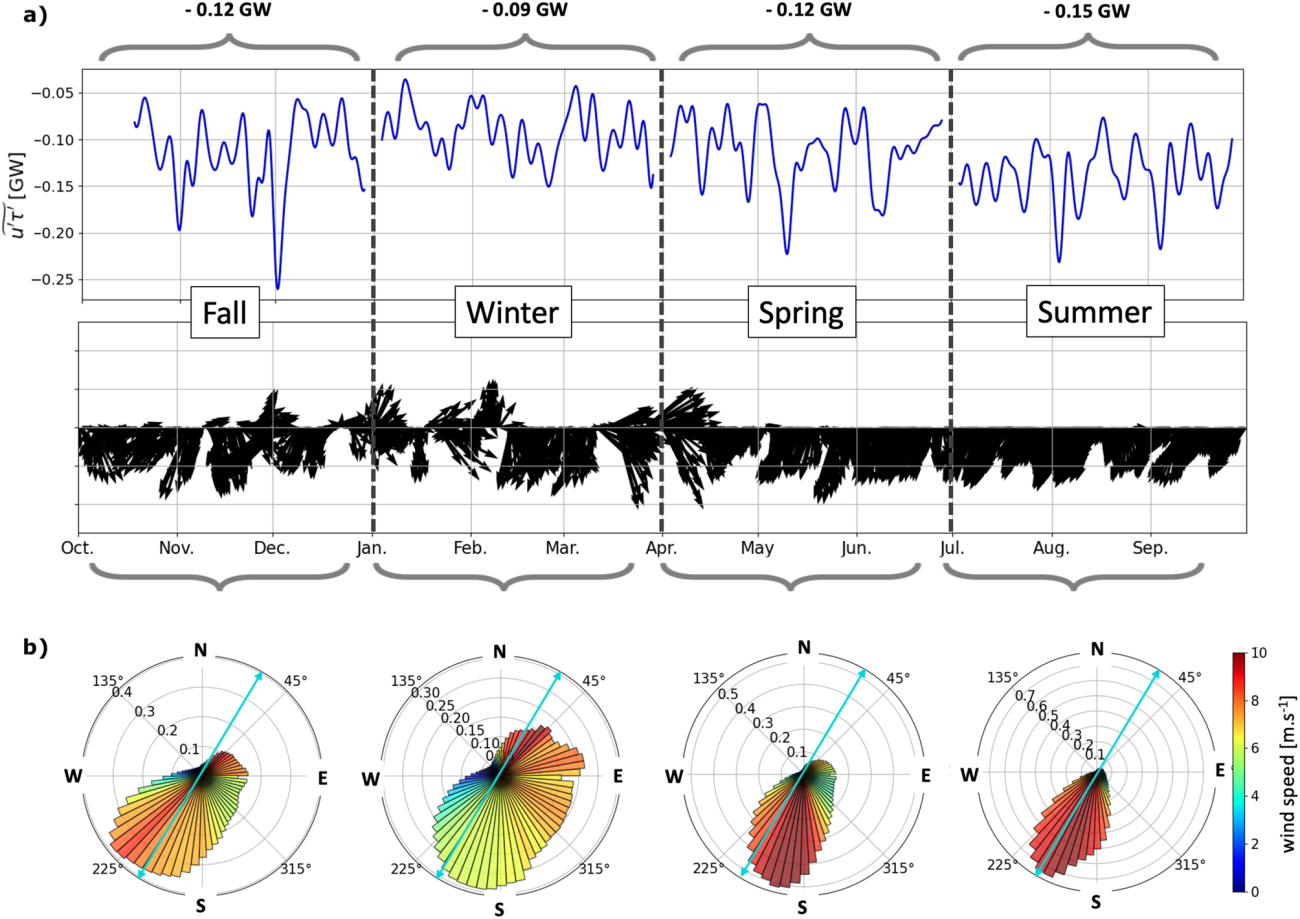
(**a**) Domain-integrated wind power as a function of time $\widetilde{u'\tau '}$, where the primes correspond to a combination of a temporal Butterworth band-pass filter with cutoff periods at 4 and 14 h, and a spatially uniform highpass filter with a cutoff scale of 180 km, where the tilde corresponds to a low-pass filter with a cutoff period of 4 days. The seasonal average is indicated above the braces. (**b**) Domain averaged wind direction. The seasonal polar PDFs are indicated below the braces. The radial axis represents the density of probability of the wind blowing in each 5$^\circ$ direction bin represented on the polar axis. The shade corresponds to the averaged wind speed for each direction bin. The blue arrow represents the averaged $u'$ polarization direction.

The energy sink associated with the wind power on internal tides varies in time and is on average larger in summer (− 0.15 GW) and smaller in winter (− 0.09 GW) (Fig. 5a). The seasonal variation in wind power appear to be correlated with the seasonal variation in the wind direction.

Indeed, the magnitude of the net average wind power for IWs is inversely correlated to the angle between the wind direction and the polarization of the surface currents of the IWs (see Fig. S3, SI). The average polarization of the surface currents associated with internal tides (frequency band 4–14 h and horizontal scales < 180 km) is in the S-SW direction (Fig. 5b). Over the USWC ocean, the wind is steadier in summer, when it is almost constantly blowing in the S-SW direction, with a probability of 0.7 for a direction of 250$^\circ$ relative to the eastward direction and an associated wind speed of 8 ms$^{-1}$ (Fig. 5b). The wind in summer is therefore frequently aligned with the direction of the internal tides radiating from the Mendocino Ridge (Fig. S4, SI). In winter, however, when the wind is oriented in many directions, it is less likely to be aligned with the internal tide currents and a less efficient energy sink is observed. As a comparison, the probability that the wind is blowing at an angle of 250$^\circ$ in winter is less than 0.3 with an averaged wind speed of 6 ms$^{-1}$.

Other parameters, such as the variability in the magnitude of the internal tides kinetic energy have been found to be less relevant for explaining the variability of the wind power at these frequencies (not shown).

#### Modal distribution

We further quantify the wind power energy sink on ITs by decomposing the currents between the contribution of the first three baroclinic modes (Fig. S5, SI). For the USWC, the negative wind power is relatively larger for higher modes (− 0.044 GW and − 0.031 GW for mode 2 and 3 respectively) than for mode 1 (− 0.026 GW), even though mode 1 has greater overall energy (1.1$\times 10^6$ GWs—Gigawatt seconds), larger by an order of magnitude than the energy contained in higher modes (Table 1). It is generally observed that mode 1 contains most of the internal tide energy. Recent global-scale estimates found 165$\times 10^6$GWs in mode 1 and 79 $\times 10^6$ GWs and 30 $\times 10^6$ GWs in mode 2 and 3 respectively for the M$_2$ constituent^48^, although these quantities may be overestimated (Buijsman et al.^4^ estimated mode 1 energy to be 84.6 $\times 10^6$ GWs). As a consequence the decay timescale associated with the negative wind power on internal tides, which is defined as the ratio between the wind power and the vertically integrated KE for each mode, is much larger for mode 1 (nearly 500 days) than for modes 2 and 3 (around 150 days, see Table 1).

**Table 1:** Comparison of the domain integrated kinetic energy ($\overline{\int \rho _0 u'^2 dz}$) and wind power $\overline{u^\prime \cdot \tau ^\prime }$ for baroclinic modes 1 to 3. The ratio between the two quantities yields the timescale needed for the wind to cancel out the kinetic energy of the internal tides, giving an idea of the internal wave killing efficiency associated with each baroclinic mode. As for Fig. 4, the prime corresponds to the F-4 h-14 h-180 km filter.

	KE $\overline{\int \rho _0 u'^2 dz}$ (GWs)	Wind power $\overline{u^\prime \cdot \tau ^\prime }$ (GW)	$|\overline{u^\prime \cdot \tau ^\prime }/\overline{\int \rho _0 u'^2 dz}|$ (days)
Mode 1	1.1 $\times$ 10$^6$	− 0.026	490
Mode 2	5.8 $\times$ 10$^5$	− 0.044	153
Mode 3	3.9 $\times$ 10$^5$	− 0.031	146

The IT killing is therefore more efficient for higher baroclinic modes. These time scales can be compared with the eddy attenuation time due to CFB, estimated to be around 250 days by Renault et al.^22^. It is about the same order of magnitude as what we find for the ITs. The “IT killing” is comparatively as efficient as the “eddy killing”.

The difference in IW damping between the different baroclinic modes can be explained by the polarization of each mode. Because IWs are polarized, their hodographs do not describe a perfect circle but an ellipse, whose longest axis direction is called the polarization direction. The polarization relations (Eq. (2)) can be derived from the linearized primitive equations, and depend on the baroclinic mode *n* through the dispersion relation (Eq. (3)):

2 $$\begin{aligned}{} & {} \begin{aligned} u&= \frac{1}{\rho _0}\left( \frac{\omega k_x + ik_y f}{\omega ^2 - f^2}\right) p \\ v&= \frac{1}{\rho _0}\left( \frac{\omega k_y - ik_x f}{\omega ^2 - f^2}\right) p, \end{aligned} \end{aligned}$$

3 $$\begin{aligned}{} & {} \omega ^2 = f^2 + c_n^2 (k_x^2 + k_y^2). \end{aligned}$$

**Figure 6:**
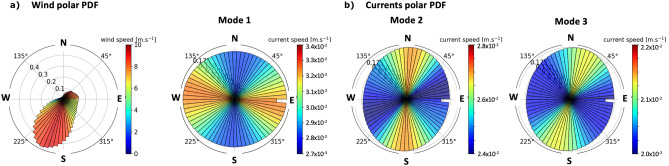
(**a**) Polar PDF for the wind velocity over 1 year; (**b**) Polar PDF for the ocean surface current velocities associated with baroclinic modes 1, 2 and 3.

The averaged polarization of the ITs in the region (mostly dominated by M$_2$) can be inferred from polar probability density functions (PDFs) of the surface currents associated with each mode. We find that mode 1 has a dominant East–West polarization, while mode 2 has a dominant North–South polarization, and mode 3 a Northeast–Southwest polarization (Fig. 6b).

The polarization of each mode is itself linked to its main propagation direction. At a given frequency, the ratio between *u* and *v* is proportional to the ratio between $k_x$ and $k_y$, the horizontal wavenumbers obtained by the dispersion relation for a given baroclinic mode n (Eq. (3)), and therefore proportional to the ratio between the eastward and northward group velocities (Eq. (4)).

4 $$\begin{aligned} \begin{aligned} c_{gx} = \frac{\partial \omega }{\partial x} = \frac{c_n^2k_x}{\omega }\\ c_{gy} = \frac{\partial \omega }{\partial y} = \frac{c_n^2k_y}{\omega }. \end{aligned} \end{aligned}$$

In the USWC region, the direction of propagation of the ITs is different for mode 1 than for higher modes. Indeed, the Mendocino Ridge is known to generate predominently internal tides with baroclinic modes $\ge 2$^43^, whose energy beams therefore propagate northward and southward of the ridge and have a signature on the surface KE of mode 2 and 3 on each side of the ridge (Fig. S6, SI). On the contrary, mode 1 is dominated by internal tide beams propagating from the Hawaiian ridge eastward into the simulation domain, as seen from the energy beams emanating from the northern and western boundary (Fig. S6, SI and Siyanbola et al.^39^). Remotely generated higher modes, which are not able to travel such large distances as mode 1, do not reach the simulation domain. This explains why the higher modes have a predominent North–South polarization ($v\gg u$), while mode 1 has a predominant East–West polarization ($u \gg v$) (Fig. 6b).

As discussed in the previous sections and SI, the magnitude of the IW damping depends on the relative angle between the current and wind velocity vectors. The polar PDF of the wind (averaged over one year) shows a mean South-Southwestward direction, with a probability of  0.45 at an angle of 250$^\circ$ relative to the East (Fig. 6a). The IT damping is therefore more important for mode 2 and 3, whose directions are almost aligned with the wind than for mode 1, whose direction has a larger angle with the wind on average (Fig. 6). This can explain the differences found in Table 1.

## Discussion

In this study, we compute the wind power on oceanic internal waves (near-inertial and super-inertial) from a numerical simulation of the northeastern Pacific along the USWC.

We show that the CFB damps IW, in a similar way as it damps mesoscale eddies (“eddy killing”; Renault et al.^22^), a process we refer to as “IW killing”. In the near-inertial band, where the wind is the source of energy for IWs, CFB reduces the net energy input by 67%. In the super-inertial band, where the barotropic tide is the main source of energy for the IW, CFB induces a net energy sink of − 0.13 GW in the region of study, representing about 8% of the internal tide generation at the topography in the region. When extrapolated to the global ocean (the simulation domain represents 0.6% of the global ocean surface), this energy sink represents − 20 GW, which is of the same order of magnitude as the energy sink induced by the wind-power on mesoscale eddies (− 28 GW^21^ to − 50 GW^23,25^). However, this estimation of the energy sink would need to be corroborated with observations, and additional high-resolution numerical simulations in other regions.

In addition, we examine the spatial and temporal variability of the “IW killing”. The seasonal variability of IW killing is due to the seasonal orientation of the wind, which is more frequently aligned with the internal tide propagation in summer, inducing a stronger energy sink in this season. This seasonal energy sink raises the question of whether the wind could be partly responsible for the seasonal cycle observed in M$_2$ internal tide energy. The modal distribution, and in particular the fact that baroclinic mode 1 internal tides are less affected than higher modes, also results from the relative direction of winds and baroclinic tides propagation. This “IW killing” effect will likely be region-dependent as the wind and internal tide propagation directions vary over the globe. To gain more perspective, it would be valuable to assess the internal tide wind-induced energy sink in other regions of strong internal tide generation such as the Hawaiian ridge^49^, the Luzon Strait^50^, the Aleutian Ridge^51^ or the mid-Atlantic Ridge^2^. Furthermore, it would be interesting to analyze the sensitivity of the energy sink induced by the CFB to the spatio-temporal resolution of the wind. It is known that the wind energy input in the near-inertial band is strongly sensitive to the temporal resolution of the wind^5^. Similarly, it would be interesting to evaluate how the spatial resolution of the wind affects the wind power over near-inertial and tidal IWs.

One of the limitation of this study is that it relies on the parametrization of the CFB in the model we use. Here, to take into account the partial reenergization of the wind by ocean currents, we use a stress correction approach^42^. This parametrization has been validated in mesoscale resolving models, but its validity at smaller scales and higher frequencies remains to be investigated. Note that in models that neglect the wind reenergization, the negative wind power estimates may be overestimated. Although we are confident that the physical mechanism underlying the IW killing is physical and should qualitatively not be dependant of this parameterization, it would be valuable to investigate this process in ocean-atmosphere coupled numerical simulations in future studies.

Our study also raises some questions about ocean mixing, for which IWs are thought to be a main energy source. Mixing and dissipation are hard to measure in the ocean or even to infer from numerical simulations because they occur at subgrid scale and therefore rely on parameterizations. Dissipation due to IWs is generally approximated using a balance between barotropic to baroclinic energy conversion and divergence of pressure fluxes^52^. In this study, we show that on average 8% of the energy converted from the barotropic tide into internal tides is dissipated by the wind. Neglecting this effect could therefore lead to overestimates of ocean mixing by the same amount. A rigorous investigation of this question would require a full energy budget, that accounts for eddy-IW energy exchanges, interaction with the topography, etc.^53^, which is beyond the scope of this paper.

Finally, this study opens some perspective for the interpretation of future satellite missions that aim to measure the wind stress and oceanic currents simultaneously (e.g., the Odysea mission^54^) and will therefore rely on our understanding of wind and current interactions.

## Methods

### Model configuration

#### Model and grid

The simulation used is this study is performed with the CROCO model (Coastal and Regional Ocean COmmunity, https://www.croco-ocean.org/)^31–33^. The CROCO model solves the primitive equations: Navier–Stokes equations in Cartesian coordinates with sigma coordinates in the vertical (terrain-following coordinates), using the Boussinesq and hydrostatic approximations with a time splitting method between the fast barotropic mode and the slow baroclinic modes. The simulation grid covers the US West Coast and encompasses the Mendocino Ridge with an horizontal grid spacing of 2 km and a 100 vertical levels with stretching parameters of $h_c=$ 350 m, $\theta _s =$ 6 and $\theta _b =$ 4.5. The initial condition is obtained from a 1 year spin up. The river-runoff is included offline as surface precipitations with a Gaussian distribution over the grid cells that fall within the range from the coast to 150 km offshore. The runoff data come from a monthly climatology from Dai et al.^55^. The model is forced at the surface (atmospheric forcing) and at the open boundaries (northern, western and southern boundaries).

#### Atmospheric forcing

The ocean is forced hourly with an atmosphere coming from a simulation of the WRF (Weather Research and Forecasting) model^40^ spanning the US West Coast at a resolution of 6 km. This simulation is nested down from an 18 km resolution simulation that spans the whole North American West Coast and that reproduces the synoptic features conditioning the smaller scale dynamics in the smaller nest. The configuration of these simulations is the same as in Renault et al.^38,56^. 10m winds, long and short wave radiations, air temperature, relative humidity and liquid precipitations are prescribed and the fluxes are computed using a bulk formula^41^ with a parameterization of the wind response to CFB with a stress correction approach^42^. Note that a high temporal resolution (1 h here) of the wind is necessary in order to accurately represent the generation of near-inertial waves^5,38^.

#### Boundary forcing

The simulation is nested from a 4km simulation of the US West Coast provided by Siyanbola et al.^39^, and which contains remotely and locally generated internal waves (including tidal forcing). The open boundary conditions (BC) between the 4 km and 2 km simulations are: Flather BC^57^ for the barotropic scheme and Specified BC^58,59^ for the baroclinic scheme. In addition, sponge layers of 50 km width and 800 m$^2$ s$^{-1}$ viscosity are implemented. These BC have been found to minimize reflections of internal waves at the open boundaries and the methodology has been validated by Siyanbola et al.^39^.

## Supplementary Information

Supplementary Information.

## Data Availability

The CROCO code is publicly available at https://www.croco-ocean.org.
