# Supplementary material for: Wind-current feedback is an energy sink for oceanic internal waves
Source: Sci Rep. 2023 Apr 11;13:5915. doi: 10.1038/s41598-023-32909-6 (PMC10090179; doi:10.1038/s41598-023-32909-6)
Supplement: Supplementary file 1 — Supplementary Information. [file 41598_2023_32909_MOESM1_ESM.pdf]

Supplementary Materials
Wind-Current feedback is an energy sink for oceanic internal waves

A. Delpech, R. Barkan, L. Renault, J. McWilliams, O. Q. Siyanbola, M. C. Buijsman, B. K. Arbic

Simulation without Current Feedback (CFB) parametrization

A similar simulation as the one presented in the main document, but with the CFB parameterization switched off was performed. As a comparison, we show the spectral wind power in Fig. S1.

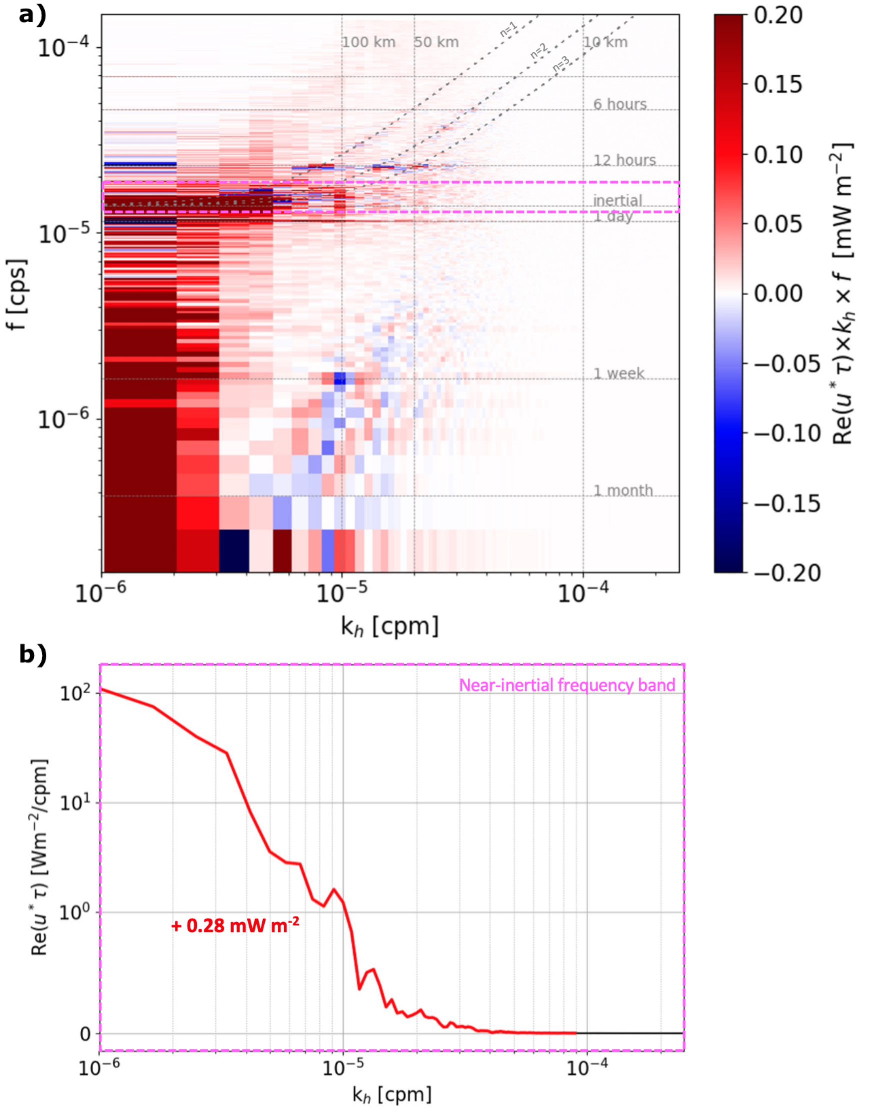

Figure S1: As Fig. 2 of the main document, but for a simulation that does not take into account the CFB. (a) Cospectrum of the wind and the surface ocean currents in horizontal wavenumber (x-axis) and frequency (y-axis), computed in the dashed box shown on Fig. 1 of main document. Positive values indicate a transfer of energy from the wind into the ocean. Negative values indicate a transfer of energy from the ocean to the wind. The dispersion relations for the first three baroclinic modes (n=1,2,3) are indicated. The purple box indicates the limits of the near-inertial frequency band. The blue box indicates the limit of the higher frequency internal wave band. (b) Cospectrum of the wind and the surface ocean currents as a function of the horizontal wavenumber integrated in the near-inertial (NI) frequency band 1.39×10^{-5} - 1.74×10^{-5} cps (periods between 16h and 20h). *This figure has been generated using Python v3.6* - `https://www.Python.org/`

Wind and surface Currents spectra

To complement the co-spectrum of wind and currents presented in Fig. 1 of the main document, we provide here the individual spectra of winds and surface currents (Fig. S2)

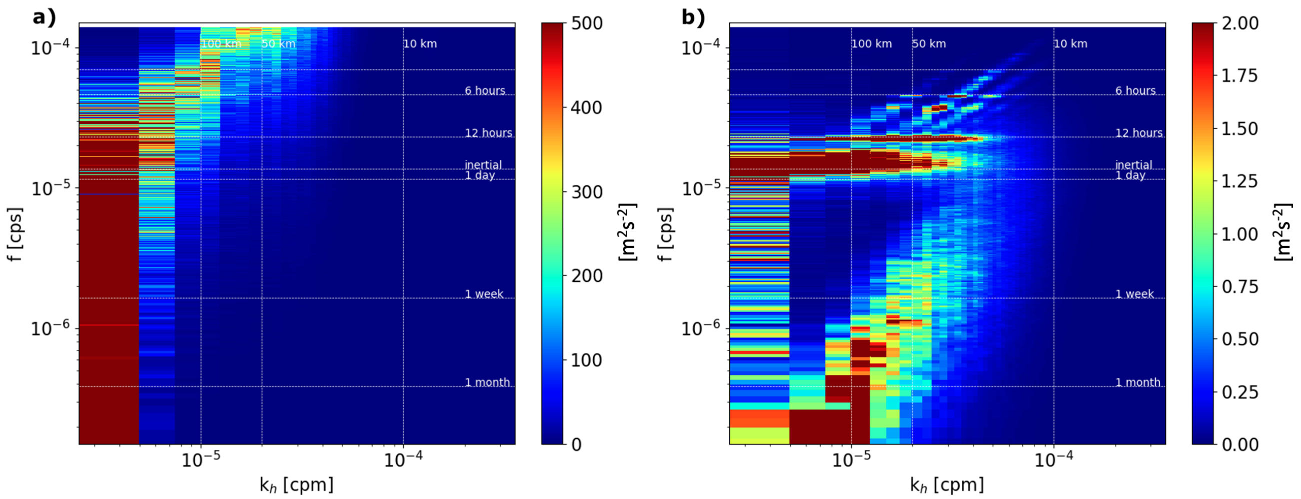

Figure S2: Horizontal wavenumber-frequency spectra of (a) winds and (b) surface currents. *This figure has been generated using Python v3.6* - `https://www.Python.org/`

Wind power bulk formula

We write $\mathbf{u}_w$ the wind vector and $\mathbf{u}_c$ the ocean current vector, with $u_w = |\mathbf{u}_w|$ and $u_c = |\mathbf{u}_c|$. If we define a normal base $(\mathbf{x}_c, \mathbf{y}_c)$ where $\mathbf{x}_c$ is aligned with $\mathbf{u}_c$ and $\mathbf{y}_c$ is the unit vector orthogonal to $\mathbf{x}_c$, then we can rewrite the two vectors in this base as:

$$\mathbf{u}_w = u_w \cos(\theta)\mathbf{x}_c + u_w \sin(\theta)\mathbf{y}_c \tag{1}$$

$$\mathbf{u}_c = u_c \mathbf{x}_c \tag{2}$$

where θ is the relative angle between the wind and the current.

The wind power can be rewritten as a function of the wind and current magnitude and of the relative angle between the two, $F(u_w, u_c, \theta) = \boldsymbol{\tau} \cdot u_c$.
In the case of a constant wind, and of an ideally polarized current, with currents oscillating along a single direction, the average wind power over one period is shown in Fig. S3. The energy sink associated with wind power is maximal when and where the wind is aligned with the currents and minimal when the wind is at right angle of the current.

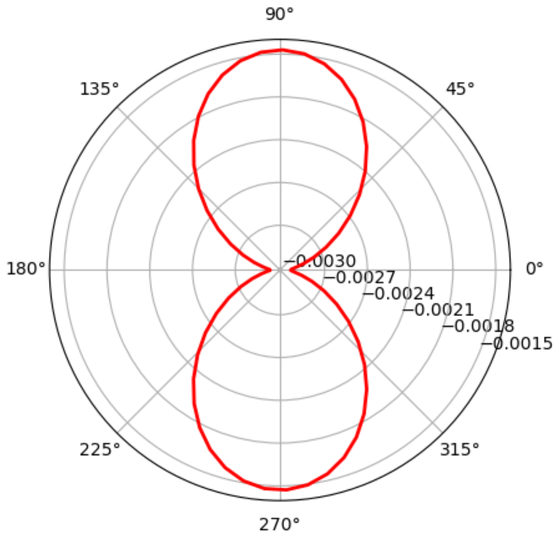

Figure S3: Wind power as a function of θ, the relative angle between the wind and the current (polar axis) for values of u_w=10 ms^{-1}, u_c=1 ms^{-1}, and a constant $C_d = 0.0012$. *This figure has been generated using Python v3.6* - `https://www.Python.org/`

Internal tide energy flux

The internal tide energy flux is computed as:

$$\overline{\mathbf{F}} = \left(\overline{\int_{-H}^{\zeta} u'p'dz}, \overline{\int_{-H}^{\zeta} v'p'dz}\right) \tag{3}$$

where H is the water depth, ζ is the sea surface height, (u', v') is the baroclinic horizontal velocity associated with internal tide and p' is the pressure perturbation associated with internal tides. The overbars correspond to a one-year average and the primes correspond to a combination of a temporal Butterworth band-pass filter with cutoff periods at 4 and 14 hours, and a spatially uniform highpass filter with a cutoff scale of 180 km.

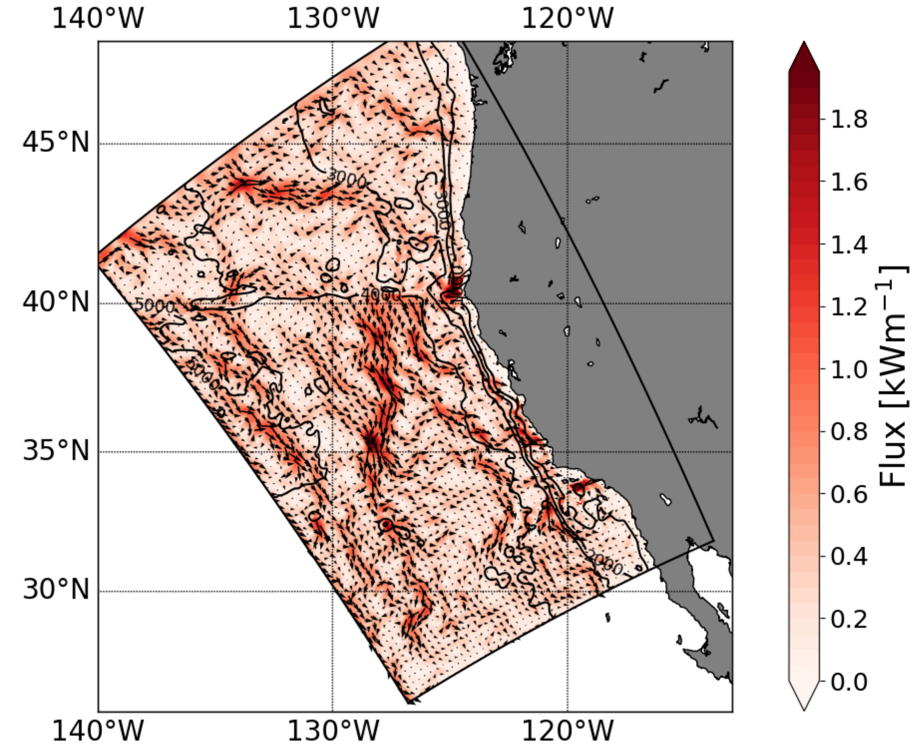

Figure S4: Intensity (color) and direction (arrows) of the depth-integrated baroclinic energy flux for internal tides. Bathymetry is in countours. *This figure has been generated using Python v3.6* - `https://www.Python.org/`

The vertically-integrated baroclinic internal tide energy flux shows a beam of internal emanating from the Mendocino Ridge at 40°N and propagating southward, as well as incoming beams propagating eastward from the northern boundary.

Baroclinic modes decomposition

The Stourm-Liouville equation for normal modes with a free surface boundary condition (e.g., Kelly, 2016) is solved at each grid point using a seasonal time averaged stratification to obtain a set of eigenfunctions. An example of these eigenfunctions is given in Fig. S5.

The decomposition of the velocity on baroclinic modes shows that on average more than 70% of the surface kinetic energy is explained by the superposition of the first 3 baroclinic modes (Fig. S6).

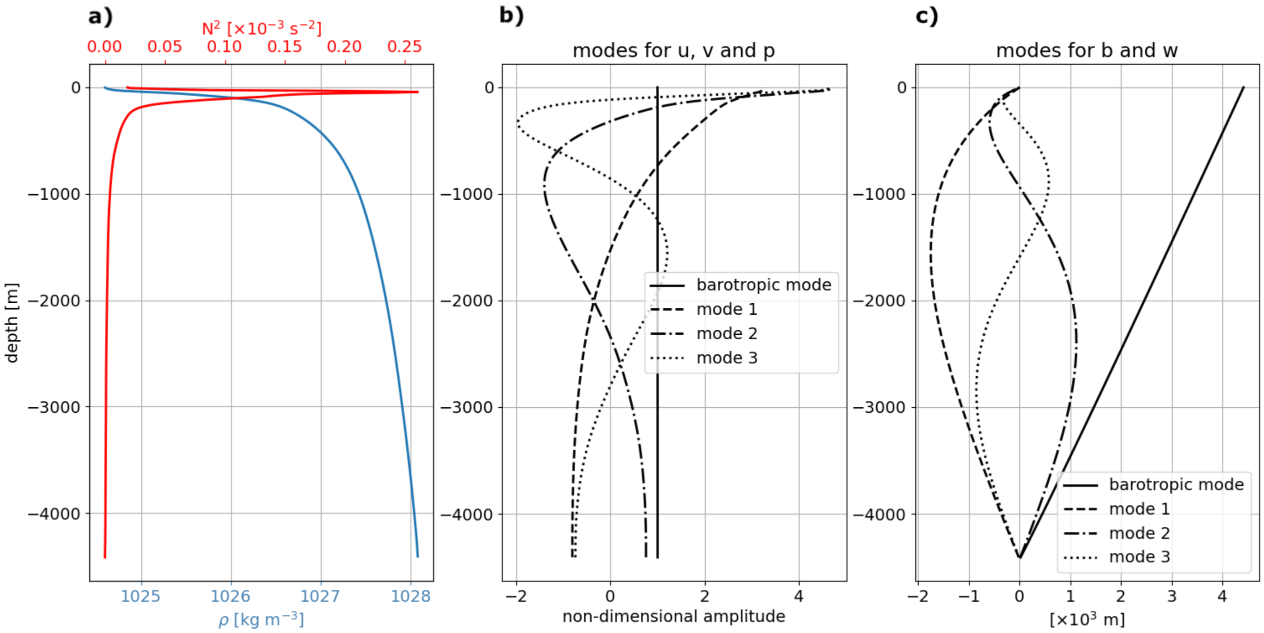

Figure S5: Example of modal decomposition at a single location. (a) time-averaged density and stratification profiles; (b-c) corresponding eigenfunctions for the barotropic and first three baroclinic modes for (b) the horizontal velocity and pressure u, v and p and (c) the buoyancy and vertical velocity b and w. *This figure has been generated using Python v3.6* - `https://www.Python.org/`

Fig. S6 also shows that mode 1 is dominated by remotely generated internal tides that propagate into the domain from the northern and western boundaries, while mode 2 and 3 are dominated by locally generated internal tides that radiate northward and southward off the Mendocino ridge.

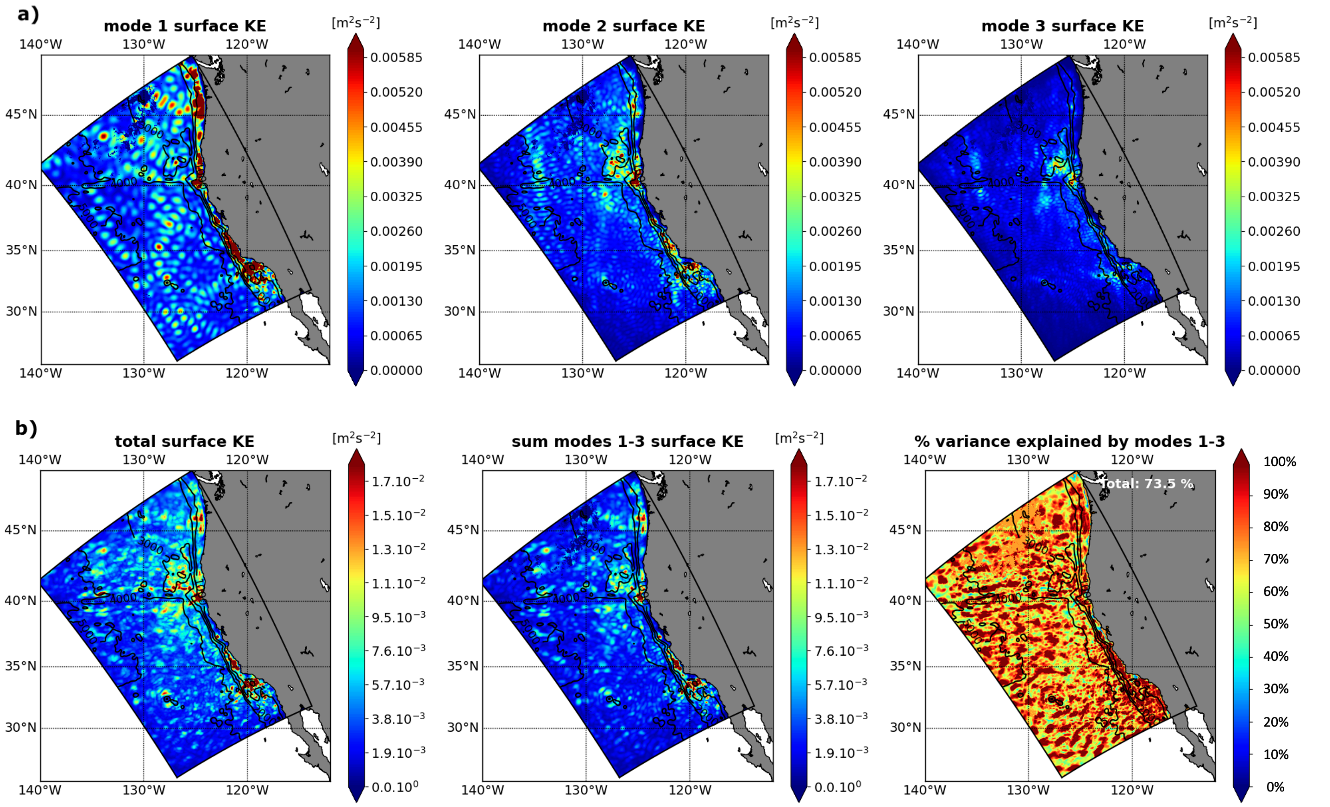

Figure S6: (a) Decomposition of surface kinetic energy (KE) on baroclinic modes 1-3. (b) Comparison of the total surface KE with the surface KE reconstructed from the contributions of baroclinic modes 1-3. The bathymetry is indicated in contours. *This figure has been generated using Python v3.6* - `https://www.Python.org/`

References

Kelly, S. M. (2016). The vertical mode decomposition of surface and internal tides in the presence of a free surface and arbitrary topography. Journal of Physical Oceanography, 46(12):3777–3788.
